# Rectal cancer with a metastasis to the thyroid gland: A case report

**DOI:** 10.1016/j.ijscr.2019.11.013

**Published:** 2019-11-18

**Authors:** Katsusuke Mori, Koji Koinuma, Hiroshi Nishino, Hisanaga Horie, Alan Kawarai Lefor, Naohiro Sata

**Affiliations:** a Department of Surgery, Jichi Medical University, 3311-1, Shimotsuke, Tochigi, 329-0498, Japan; b Department of Otolaryngology/Head and Neck Surgery, Jichi Medical University, 3311-1, Shimotsuke, Tochigi, 329-0498, Japan

**Keywords:** CT, computed tomography, PET, positron emission tomography, Thyroid metastasis, Colorectal cancer, Case report

## Abstract

**Introduction:**

Metastases to the thyroid gland in patients with colorectal cancer are uncommon. We report a patient with rectal cancer who developed a metastasis to the thyroid gland.

**Presentation of case:**

The patient was a 45-year-old female five years status post rectal cancer resection. A thyroid lesion was detected on PET-CT scan with synchronous lung metastases. After pulmonary resection, a partial thyroidectomy was performed and pathological examination with immunohistochemical staining confirmed that the lesion was a metastasis from previous rectal cancer. She is free from recurrence two years after thyroid surgery.

**Discussion:**

Colorectal metastases to the thyroid gland are usually seen with widespread disease, often with lung and liver metastases. The overall outcomes of previously reported patients with thyroid metastases were extremely poor, with most patients dying within months of diagnosis. Careful attention should be given to other sites of metastatic disease including the thyroid gland during postoperative follow-up. PET scan may be helpful to establish the diagnosis.

**Conclusion:**

Treatment decisions must be individualized, and depend on the presence of systemic disease. Selected patients may benefit from resection of metastases, and PET scan may be useful to identify patients who will benefit from resection.

## Highlights

• Colorectal cancer metastases to the thyroid gland are extremely rare.
• Although most patients with thyroid metastases have widespread disease, selected patients may benefit from resection.
• PET scan may help identify patients who will benefit from resection.

## 1 Introduction

There are few reports of metastases to the thyroid gland [1]. Thyroid gland metastases from kidney, breast, melanoma and lung primary tumors have been reported, with few reports of gastrointestinal malignancies [[2], [3], [4]]. Colorectal carcinoma metastatic to the thyroid gland is particularly rare.

We report a patient with rectal cancer with lung metastases who was diagnosed with a thyroid metastasis five years after rectal cancer resection. To the best of our knowledge, there is only one other patient with more than two years survival without recurrence. This patient is presented with a review of the literature for colorectal cancer metastases to the thyroid gland.

This work is reported in accordance with the SCARE criteria [5].

## 2 Presentation of case

A 45-year-old female underwent a low anterior resection for rectal cancer, and the pathological diagnosis was tubular adenocarcinoma, pT3, N0, M0, ly0, v1, Stage II. She did not receive adjuvant chemotherapy. Three years after resection of the primary tumor, she was diagnosed with lung metastases in the left upper and lower lobes. Positron emission tomography (PET) scan showed two areas of abnormal fluoro-2 deoxy-D-glucose accumulation in the lung with no evidence of other distant metastases. Both lesions were resected with video-assisted thoracoscopic surgery, and eight course of CapeOX (capecitabine and oxaliplatin) chemotherapy was given.

**Fig. 1:**
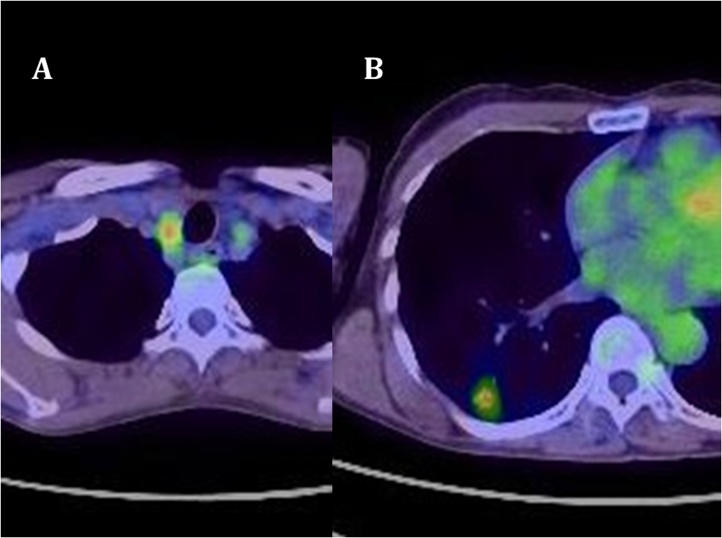
Abnormal uptake areas of fluoro-2 deoxy-D-glucose are seen in the right lobe of the thyroid gland (A) and right lower lung (B) on positron emission tomography – computed tomography imaging.

**Fig. 2:**
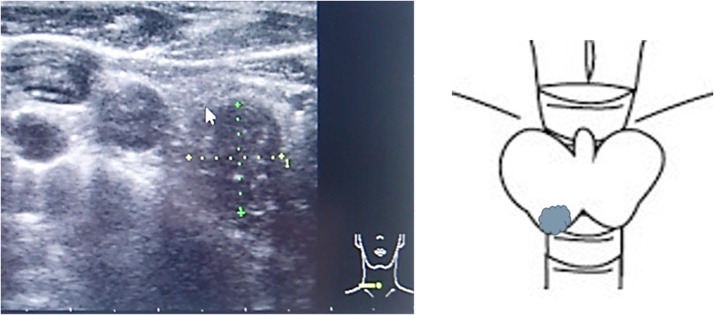
A 12 mm mass is seen in the right lower thyroid lobe with ultrasonography.

**Fig. 3:**
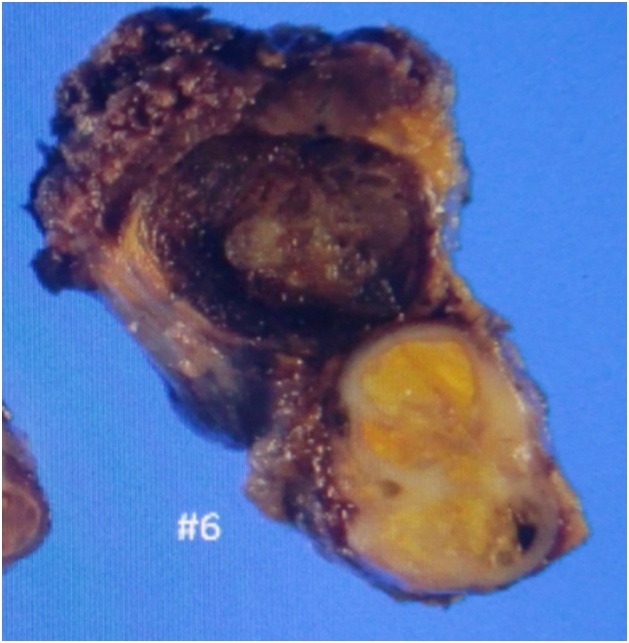
A yellowish thyroid tumor is seen in the resected specimen.

**Fig. 4:**
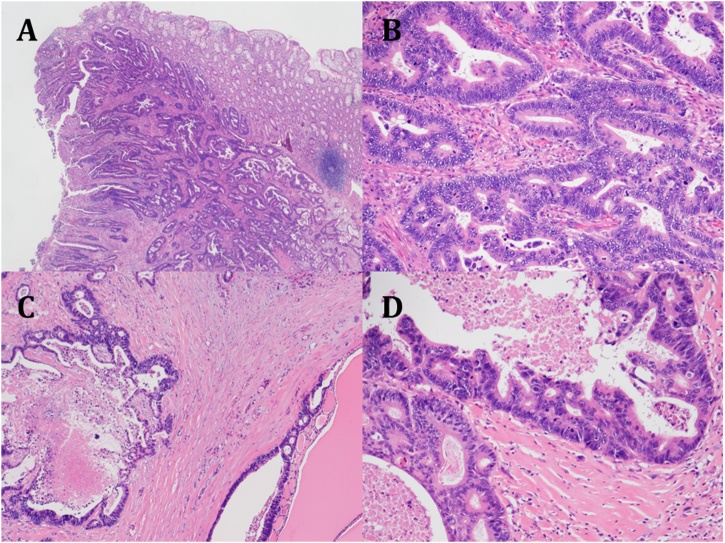
Pathology of the primary rectal cancer (A; ×100, B; ×400, H&E) and thyroid tumor (C; ×100, D; ×400, H&E).

**Fig. 5:**
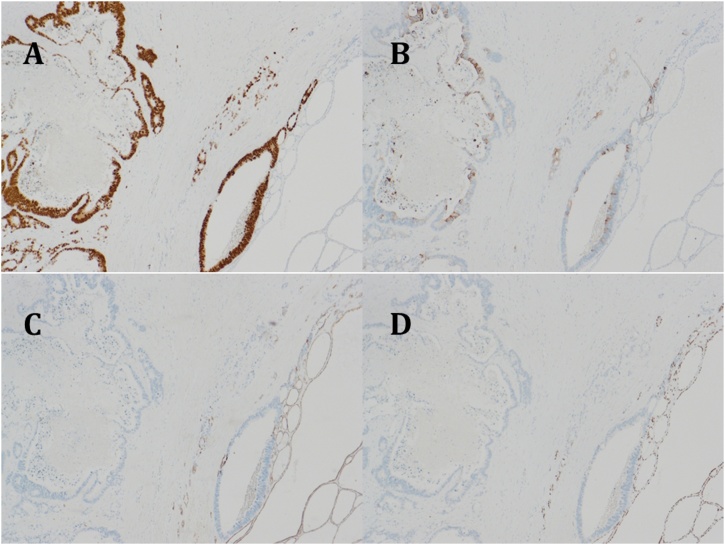
Immunohistochemical examination shows the thyroid tumor to be positive for CK20 (A; ×100), CDX-2 (B; ×100), and negative for CK7 (C; ×100), TTF (D; ×100).

Two years after pulmonary resection, the serum carcinoembryonic antigen level was increasing, and computed tomography (CT) scan of the chest showed a mass in the right lower lung. A lesion was also seen in the right lobe of the thyroid gland on CT scan. PET scan showed abnormal accumulation in both the lung and thyroid gland (Fig. 1). A 12 mm thyroid mass was also seen with ultrasonography (Fig. 2) and fine needle aspiration cytology was not diagnostic. The thyroid mas was thought to be a primary thyroid lesion, because metastasis from the previous rectal cancer was considered highly unlikely. Partial right lung resection by video-assisted thoracoscopic surgery was performed. Subsequently, a right thyroidectomy with lymph node dissection was performed (Fig. 3). Postoperative course was uneventful. Pathologic examination showed adenocarcinoma with no lymph node involvement (Fig. 4). Immunohistochemical examination showed positive for CK20, CDX-2, and negative for CK7, TTF (Fig. 5), which was compatible with metastasis from the previously resected rectal cancer. No adjuvant chemotherapy was given, and she remains free of recurrent disease two years after thyroid gland resection, eight years after resection of the primary rectal cancer.

## 3 Discussion

Metastases to the thyroid gland are uncommon. The incidence of thyroid metastases among patients with colorectal cancer is very low, and Lievre et al. reported 6 patients (0.1 %) with thyroid metastases of 5862 patients with colorectal cancer from a single institution [6].

Two studies reviewed 31 and 35 patients with thyroid metastases from colorectal cancer, with ages ranging from 34 to 85 year and two-thirds being female [7,8]. The majority of the colorectal cancers were left-sided with half in the rectosigmoid. Metastases to the thyroid were diagnosed from 0 months (synchronous) to 8 years after colorectal resection. The lung was the most frequent other metastatic site in these patients (either synchronous or metachronous), in about 60 % of the patients with thyroid metastases. The present patient had a Stage II rectal cancer and underwent two pulmonary resections with the thyroid metastasis diagnosed five years after rectal cancer resection. Most of the reported patients have had lung and/or liver metastases, favoring a hematogenous metastatic route. Most patients reported had widespread disease including lung and/or liver metastases, with an unfavorable prognosis. However, there are some patients with thyroid metastases without other distant lesion which could be explained by spread of tumor cells to the inferior vena cava through the vertebral vessels [9].

The overall outcomes of previously reported patients with thyroid metastases were extremely poor, with most patients dying within months of diagnosis. Some patients had a survival of two years or more, however, most of them had widespread disease and were treated with chemotherapy or radiation. Only one previously reported patient had prolonged survival without further recurrence [7]. To the best of our knowledge, there are the only two patients with long-term survival without recurrence.

Fine needle aspiration cytology/biopsy are useful to diagnose thyroid masses [10,11]. Fine needle aspiration biopsy in combination with immunohistochemical staining could distinguish between metastatic and primary cancer [[12], [13], [14]]. Recently, PET scan is increasingly used for the diagnosis, preoperative staging and follow-up of patients with malignancies [15]. A number of investigators have shown that primary and metastatic thyroid cancers from many organs including colorectum can be seen on PET scan [12,16,17]. According to the authors, increased thyroid fluoro-2 deoxy-D-glucose uptake in the PET scan can be the first and only sign of thyroid neoplasm. They also reported difficulty in distinguishing between primary and metastatic thyroid tumors. Fine needle aspiration should be used for preoperative diagnosis of thyroid masses but was non-diagnostic in the present patient.

The ideal treatment modality should be determined considering the presence or absence of metastases to other sites, the patient’s general condition, and the presence or absence of local symptoms. Most patients with thyroid metastases from colorectal cancer had widespread disease with short overall survival.

## 4 Conclusion

Although patients with thyroid metastases from colorectal cancer usually have widespread disease with a poor overall survival, selected patients will benefit from resection. PET scan may be helpful in the diagnosis of thyroid metastases, especially in the presence of lung or liver metastasis. The timing of thyroid resection must be carefully determined considering control of other metastatic sites and may lead to a favorable outcome.

## Sources of funding

This work did not receive any specific grant from funding agencies in the public, commercial, or not-for-profit sectors.

## Ethical approval

This is a case report and it didn’t require ethical approval from ethics committee according to our institution.

## Consent

Witten consent was obtained from the patient for publication of this case reports and any accompanying images.

## Author contribution

Mori K and Koinuma K; conception of study, acquisition, analysis and interpretation of data.

Koinuma K; Drafting the article.

Nishino H, Sadatomo A, Naoi D, Tahara M, Ito H, Inoue Y, Kono Y; Management of case.

Koinuma K, Horie H, Lefor A, Sata N; Critical revision of article and final approval of the version to be submitted.

## Registration of research studies

This is a case report study.

## Guarantor

Koji Koinuma.

## Provenance and peer review

“Editorially reviewed, not externally peer-reviewed”.

## Declaration of Competing Interest

All authors have no conflicts of interest.
